# The risk of cesarean delivery after labor induction among women with prior pregnancy complications: a subgroup analysis of the AFFIRM study

**DOI:** 10.1186/s12884-019-2615-x

**Published:** 2019-11-29

**Authors:** Leslie Skeith, Grégoire Le Gal, Johanna I. P. de Vries, Saskia Middeldorp, Mariëtte Goddijn, Risto Kaaja, Jean-Christophe Gris, Ida Martinelli, Ekkehard Schleußner, David Petroff, Nicole Langlois, Marc A. Rodger, for the AFFIRM investigators

**Affiliations:** 1 Division of Hematology and Hematological Malignancies, Department of Medicine, University of Calgary, C210 Foothills Medical Centre, 1403 29th Street, NW, Calgary, Alberta T2N 2T9 Canada; 2 Clinical Epidemiology Program, Ottawa Hospital Research Institute, Ottawa, ON Canada; 3 Division of Hematology, Department of Medicine, University of Ottawa, Ottawa, ON Canada; 4 Department of Obstetrics and Gynecology, VU Medical Center, Amsterdam, the Netherlands; 5 Academic Medical Center, Department of Vascular Medicine, Amsterdam, the Netherlands; 6 Academic Medical Center, Center for Reproductive Medicine, Department of Obstetrics and Gynecology, Amsterdam, the Netherlands; 7 Department of Medicine, University of Turku and Turku University Hospital, Turku, Finland; 8 Department of Hematology, Nimes University Hospital and University of Montpellier, Montpellier, France; 9 A. Bianchi Bonomi Hemophilia and Thrombosis Center, Fondazione IRCCS Ca’ Granda-Ospedale Maggiore Policlinico, Milan, Italy; 10 Department of Obstetrics and Gynecology, Jena University Hospital Friedrich Schiller University, Jena, Germany; 11 Clinical Trial Centre, University of Leipzig, Leipzig, Germany; 12 Department of Obstetrics and Gynecology, University of Ottawa, Ottawa, ON Canada

**Keywords:** Induced labor, Cesarean section, Pre-eclampsia, Low-molecular-weight heparin

## Abstract

**Background:**

To determine the risk of cesarean delivery after labor induction among patients with prior placenta-mediated pregnancy complications (pre-eclampsia, late pregnancy loss, placental abruption or intrauterine growth restriction).

**Methods:**

The AFFIRM database includes patient level data from 9 randomized controlled trials that evaluated the role of LMWH versus no LMWH during pregnancy to prevent recurrent placenta-mediated pregnancy complications. The primary outcome of this sub-study was the proportion of women who had an unplanned cesarean delivery after induction of labor compared to after spontaneous labor.

**Results:**

There were 512 patients from 7 randomized trials included in our sub-study. There was no difference in the risk of cesarean delivery between women with labor induction (21/148, 14.2%) and spontaneous labor (79/364, 21.7%) (odds ratio (OR) 0.60, 95% CI, 0.35–1.01; *p* = 0.052). Among 274 women who used LMWH prophylaxis during pregnancy, the risk of cesarean delivery was lower among those that underwent labor induction (9.8%) compared to spontaneous labor (22.4%) (OR 0.38, 95% CI, 0.17–0.84; *p* = 0.01).

**Conclusions:**

The risk of cesarean delivery is not increased after labor induction among a higher risk patient population with prior pregnancy complications. Our results suggest that women who receive LMWH during pregnancy might benefit from labor induction.

## Background

Induction of labor occurs in one out of five pregnancies and may be due to maternal, fetal or elective indications [1, 2]. While induction of labor can reduce maternal and fetal risk in patients with pre-eclampsia or intrauterine growth restriction, it is unclear if induction of labor is associated with an increased rate of cesarean delivery (CD) in these patients, an intervention that carries its own risks [1, 2]. By better understanding the risk of CD after an induction of labor in high-risk patients, clinicians and policymakers can inform future practice and improve patient care.

The ‘dogma’ that induction of labor leads to an increased risk of CD was controversial, and has been recently challenged [1, 3]. A limitation in previous studies was the lack of appropriate control group (spontaneous labor versus expectant management) and confounding factors resulting from indications for induction of labor [4]. There was no increased risk of CD reported after controlling for maternal and fetal indications of induction using multivariate analyses or propensity score matching in cohort and large database studies [5–7]. Previous randomized trials and meta-analyses evaluating patients undergoing induction of labor versus expectant management show no increased risk of CD, with the majority of trials including patients who were electively induced post-dates [1–3, 8, 9]. The ARRIVE trial randomized 3062 low-risk nulliparous women to labor induction at 39 weeks versus expectant management [10]. While there was no difference in the composite perinatal outcome, this trial has the potential to be practice changing because there was a significantly lower rate of CD and hypertensive disorders of pregnancy reported in the induction group [10, 11].

Medical indications for induction are heterogeneous, and may influence the risk of CD to varying degrees. Based on a limited number of studies, there is likely no increased risk of cesarean delivery after induction of labor among patients with pre-eclampsia or chronic hypertension, with improved maternal outcomes [12–16]. Women who have a previous history of late pregnancy loss, pre-term birth, or delivery of a small-for-gestational-age (SGA) infant are at increased risk of developing complications such as a stillbirth in a future pregnancy [17]. While preventing recurrent stillbirth is a possible indication for induction of labor, little is known about the associated risk of CD after induction of labor in these higher risk patient populations [18].

In some countries low-molecular-weight heparin (LMWH) use during pregnancy is considered a maternal indication for induction. Prophylactic-dose LMWH is recommended to stop at least 10–12 h before epidural analgesia and therapeutic-dose LMWH is recommended to stop at least 24 h before a planned delivery to minimize peripartum bleeding and improve the chance of receiving epidural anesthesia during labor [19]. It is possible that women who are induced because they are on LMWH may be at increased risk of CD, so this subgroup requires further study.

The AFFIRM project is an individual patient level database of nine randomized trials (*n* = 1048) that pooled data from patients with previous placenta-medicated pregnancy complications (pre-eclampsia, birth weight < 10th percentile, placental abruption, or late pregnancy loss). The results of an AFFIRM meta-analysis showed no difference in the risk of placenta-mediated pregnancy complications with or without the use of LMWH prophylaxis given during pregnancy, with details described elsewhere [20]. The majority of the trials in AFFIRM collected data about the labor and delivery mode, so is a rich source of information about the risk of induction of labor in a higher risk pregnant population where confounding factors are present.

The aim of this study was to determine the risk of CD after induction of labor among women with previous placenta-mediated pregnancy complications, including better understanding the role of LMWH prophylaxis on the risk of CD after induction of labor.

## Methods

### Data collection

The eligibility criteria for entry into the AFFIRM database included women with a history of one of more of the following: pre-eclampsia, gestational age adjusted birth weight < 10th percentile, clinical diagnosis of placental abruption leading to delivery, 2 pregnancy losses > 12 weeks gestation or 1 pregnancy loss > 16 weeks gestation [20]. Definitions of pre-eclampsia, severe-pre-eclampsia, birth of a small-for-gestational age (SGA) and neonatal death are described in detail elsewhere [20, 21]. Patients in the AFFIRM database were excluded from this sub-study if their current pregnancy ended in pregnancy loss, there was no labor data collected, there was no trial of labor because of an elective, unscheduled or emergent CD, or if an individual study principal investigator did not consent to data use for this study.

### Data analysis

Demographic information according to induction of labor and spontaneous labor was reported, including participant age at trial enrollment, gravida, previous live births and pregnancy losses, body mass index (BMI), race, current or recent smoker having quit in the last year, previous venous thromboembolism (VTE: proximal deep vein thrombosis (DVT) and/or pulmonary embolism (PE)), and use of LMWH prophylaxis during pregnancy. Multiple gestation, gestational age at delivery and previous and current placenta-medicated pregnancy complications, including pre-term delivery < 37 weeks of gestation, pre-eclampsia, placental abruption requiring delivery, and SGA < 10th percentile, were reported. Data about the Bishop score and the type and duration of induction of labor was not available. Data were summarized as means with ranges and standard deviations (SD) for continuous variables, and frequencies with percentages for categorical variables.

The primary outcome was the proportion of patients undergoing a CD after induction of labor, compared to after spontaneous labor. Secondary outcomes included complications of VTE, peripartum major bleeding, peripartum minor bleeding, postpartum major bleeding, neonatal mortality and maternal mortality. We also completed a planned subgroup analysis to evaluate the effect of LMWH use on the proportion of women undergoing cesarean delivery after induction of labor. Outcomes were reported using the Pearson’s chi-square test or Fisher’s exact test as appropriate, with odds ratios and 95% confidence intervals reported, with *p* values < 0.05 significant. Proportions were reported using Wilson’s score method, with 95% confidence intervals reported. A post-hoc analysis was completed to evaluate if there was a difference in gestational age between the LMWH and no LMWH groups and the induction of labor and spontaneous labor groups. Covariates were not included in the analysis when > 10% of the data was missing.

Cesarean delivery included any unplanned or emergency CD that took place after a trial of labor. If there was no trial of labor then these CD were excluded. VTE was defined as objectively confirmed DVT or PE based on compression ultrasonography, lung ventilation/perfusion or computed tomography pulmonary angiography (CTPA). Peripartum major bleeding was defined as hemorrhage occurring after the onset of labor or start or surgical delivery and within 24 h postpartum with at least one of the following: necessitating a surgical procedure, associated with a fall in hemoglobin of 4 g/dL or more, requirement for transfusion of 2 or more units of red blood cells or whole blood, estimated peripartum blood loss > 1000 ml, or considered to have contributed to maternal death. Peripartum minor bleeding was defined as hemorrhage occurring after the onset of labor or start of surgical delivery and within 24 h postpartum but does not meet any criterion above and estimated peripartum blood loss is between 500 and 1000 ml^20^. Postpartum major bleeding was defined as hemorrhage occurring between 24 h and 6 weeks postpartum with at least one of the following criteria: associated with a fall in hemoglobin of 2 g/dL or more, requirement for transfusion of 2 or more units of red blood cells or whole blood, symptomatic bleeding occurring in a critical site: intracranial, intraspinal, intraocular, pericardial, intra-articular, intramuscular with compartment syndrome, retroperitoneal, or considered to have contributed to maternal death [22]. Data were analyzed using SPSS software (IBM SPSS Statistics, Version 24.0, Armonk, NY).

## Results

**Fig. 1:**
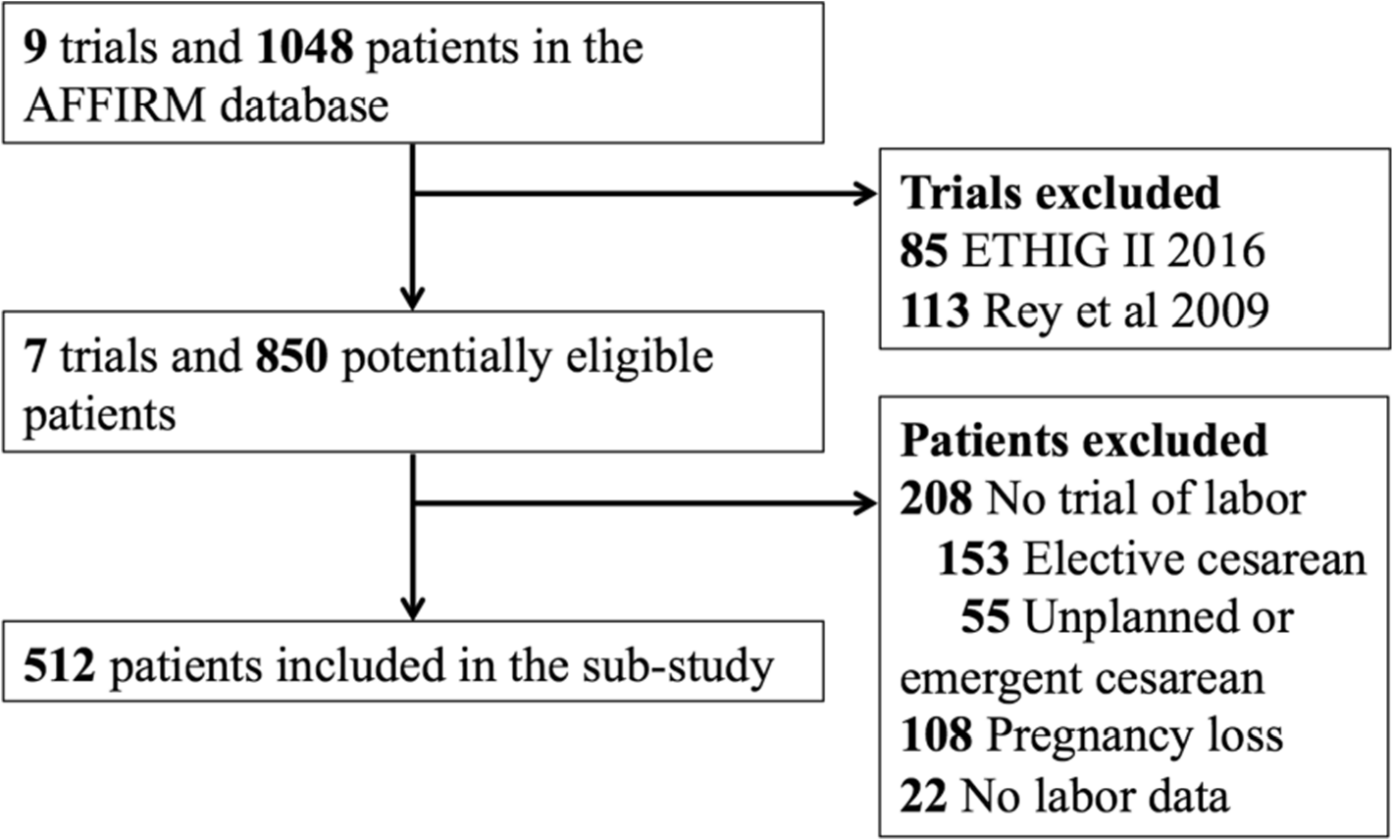
Study Flow Diagram

There were 512 patients from seven randomized trials included in this sub-study among 1048 AFFIRM patients in the database [23–29]. Participants from the ETHIG II trial were excluded because no induction data was collected (*n* = 85) [30] and one of the study investigators did not consent the use of individual trial data (*n* = 113) [31]. Additional participants were excluded because of missing labor data (*n* = 22), current pregnancy ending in pregnancy loss (*n* = 108), and no trial of labor because of elective CD (*n* = 153) or an unscheduled or emergency CD (*n* = 55) (Fig. 1).

The mean age of participants was 30.3 years old, with a mean of 2.6 pregnancies and 1.0 prior live births reported. Prior pregnancies were complicated by preterm delivery (82.4%), pre-eclampsia (61.3%), placental abruption requiring delivery (29.5%) and SGA neonates <10th percentile (28.0%) (Table 1). The placenta-mediated pregnancy complications reported in the AFFIRM study pregnancy included pre-eclampsia (6.1%), placental abruption requiring delivery (0.40%) and SGA neonate <10th percentile (14.5%) (Table 1). Demographic data according to labor type is reported in Table 1. There were 50% of women in the induction of labor group who had a prior pregnancy loss and 28.6% who had a pregnancy loss > 20 weeks, compared to 19.8 and 9.0% in the spontaneous labor group, respectively (Table 1). The proportion of participants who underwent induction of labor according to individual trial varied (Table 2).

**Table 1:** Demographic and reproductive information in the total population and according to labor type

	Total population (*n* = 512)	Induction of labor (*n* = 148)	Spontaneous labor (*n* = 364)	Comparison between labor groups
Female age: Mean (range, SD)	30.3 (19.0–43.5, 5.0)	31.45 (19.7–43.6,4.9)	29.8 (19.0–42.0, 5.0)	*p* = 0.001
Prior live births: Mean (range, SD)	1.0 (0–3, 0.5)	0.99 (0–3, 0.7)	0.99 (0–3, 0.4)	*p* = 0.93
Gravida: Mean (range, SD)	2.6 (2–11, 1.2)	3.1 (2–8, 1.5)	2.4 (2–11, 1.0)	*p* < 0.001
Multiple gestation: *n* (%)	2 (0.4)	1 (0.7)	1 (0.3)	*p* = 0.51
Gestational age at delivery: Mean (range, SD)	37.9 (20.0–44.3, 2.6)	38.8 (34.7–42.4, 1.5)	37.5 (20.0–44.3, 2.8)	*p* < 0.001
Recent or current smoker^a^: *n* (%)	46 (10.3)	21 (15.4)	25 (7.1)	*p* = 0.005
BMI: Mean (range, SD)	25.3 (17.2–65.2, 4.6)	26.3 (18.0–65.2, 5.5)	25.0 (17.2–47.6, 4.1)	*p* = 0.03
Prior VTE: *n* (%)	2 (0.4)	1 (0.7)	1 (0.3)	*p* = 0.503
Race: *n* (%)				
Caucasian	447 (87.3)	121 (81.8)	326 (89.6)	*p* = 0.016
Asian	16 (3.1)	8 (5.4)	8 (2.2)	*p* = 0.059
Black	17 (3.3)	4 (2.7)	13 (3.6)	*p* = 0.619
Other	14 (2.7)	7 (4.7)	7 (1.9)	*p* = 0.078
Unknown	18 (3.5)	8 (5.4)	10 (2.7)	–
Placenta-mediated pregnancy complications in a previous pregnancy				
Pre-term delivery < 37 wks: *n* (%)	420 (82.4)	106 (72.1)	314 (86.5)	*p* < 0.001
Pre-eclampsia: *n* (%)	314 (61.3)	80 (54.1)	234 (64.3)	*p* = 0.031
Placental abruption: *n* (%)	151 (29.5)	18 (12.2)	133 (36.5)	*p* < 0.001
SGA < 10th percentile: *n* (%)	143 (28.0)	44 (29.7)	99 (27.3)	*p* = 0.587
Prior pregnancy loss: *n* (%)	146 (28.5)	74 (50.0)	72 (19.8)	*p* < 0.001
Mean prior pregnancy losses (range, SD)	0.57 (0–9, SD 1.2)	1.08 (0–6, SD 1.4)	0.36 (0–9, SD 1.0)	*p* < 0.001
Prior late loss > 20 weeks: *n* (%)	68 (14.2%)	36 (28.6%)	32 (9.0%)	*p* < 0.001
Placenta-mediated pregnancy complications during the AFFIRM study pregnancy				
Pre-term delivery < 37 weeks: *n* (%)	141 (27.5)	10 (6.8)	131 (36.0)	*p* < 0.001
Pre-eclampsia: *n* (%)	31 (6.1)	15 (10.1)	16 (4.4)	*p* = 0.014
Placental abruption: *n* (%)	2 (0.40)	0 (0)	2 (0.5)	*p* = 0.366
SGA < 10th percentile: *n* (%)	74 (14.5)	23 (15.5)	51 (14.0)	*p* = 0.742
LMWH use during pregnancy: *n* (%)	274 (53.5)	82 (55.4)	192 (52.7)	*p* = 0.585

SGA: Small-for-gestational-age; BMI: Body mass index; VTE: venous thromboembolism, including proximal deep vein thrombosis or pulmonary embolism

^a^Recent smoker is defined as quitting within 1 year

**Table 2:** Proportion of women who underwent induction of labor and spontaneous labor according to individual trials

Trial	Induction of labor *n* (%)	Spontaneous labor *n* (%)	Total
TIPPS [23]	46 (66.7)	23 (33.3)	69
FRUIT [28]	48 (50.5)	47 (49.5)	95
HAPPY [29]	19 (29.2)	46 (70.8)	65
HABENOX [25]	6 (85.7)	1 (14.3)	7
NOH AP [26]	3 (2.9)	102 (97.1)	105
NOH PE [27]	19 (12.2)	137 (87.8)	156
ALIFE [24]	7 (46.7)	8 (53.3)	15
Total	148	364	512

In the primary analysis, there was no significant difference in the risk of CD between induction of labor (21/148, 14.2%) and spontaneous labor (79/364, 21.7%) (odds ratio (OR) 0.60, 95% CI, 0.35–1.01; *p* = 0.052) (Table 3). The gestational age at delivery for the induction of labor group was 38.8 weeks gestation, compared to 37.4 weeks gestation in the spontaneous labor group (*p* < 0.01).

**Table 3:** Complications associated with induction of labor compared to spontaneous labor

	Induction of labor *n*/*N* (%)	Spontaneous labor *n*/*N* (%)	Odds Ratio (95% CI), *p* value
Cesarean delivery	21/148 (14.2)	79/364 (21.7)	0.60 (0.4–1.0), *p* = 0.052
LMWH use	8/82 (9.8)	43/192 (22.4)	0.38 (0.2–0.8), *p* = 0.014
No LMWH use	13/66 (19.7)	36/172 (20.9)	0.93 (0.5–1.9), *p* = 0.833
VTE	0/141 (0)	0/356 (0)	–
Peripartum major bleeding	6/145 (4.1)	2/363 (0.6)	7.79 (1.6–39.0), *p* = 0.003^a^
Peripartum minor bleeding	18/145 (12.4)	16/363 (4.4)	3.07 (1.5–6.2), *p* = 0.001
Postpartum major bleeding	2/148 (1.4)	0/364 (0)	--, *p* = 0.083^a^
Neonatal mortality	1/142 (0.7)	5/356 (1.4)	0.50 (0.1–4.3), *p* = 0.680^a^
Maternal mortality	0/148 (0)	0/364 (0)	–

-- Unable to calculate an odds ratio

^a^Statistics based on Fisher’s exact test

Out of 512 participants, 274 (53.5%) were randomized to receive LMWH prophylaxis during pregnancy. The proportion of women who underwent an induction of labor and used LMWH prophylaxis during pregnancy was 29.9% (82/274, 95% CI, 24.81–35.60) compared to 27.7% (66/238, 95% CI, 22.43–33.74) who did not use LMWH. Among the 274 women who used LMWH prophylaxis during pregnancy, the risk of CD was lower among those that underwent an induction of labor (9.8%) compared to spontaneous labor (22.4%) (OR 0.38, 95% CI, 0.17–0.84; *p* = 0.01). Among the 238 women who did not use LMWH prophylaxis during pregnancy, there was no significant difference in the risk of CD among those that underwent an induction of labor (13/66, 19.7%) compared to spontaneous labor (36/172, 20.9%) (OR 0.93, 95% CI, 0.46–1.88; *p* = 0.83) (Table 3). The gestational age at delivery was 38.0 weeks in the LMWH group, compared to 37.7 weeks in the no LMWH group (*p* = 0.26).

There was an increased risk of peripartum major and minor bleeding among women who received an induction of labor compared to spontaneous labor (Table 3). Among women who received LMWH during pregnancy, two (2.5%) had peripartum major bleeding and 11 (13.8%) had peripartum minor bleeding in the induction of labor group, compared to one (0.5%) and one (0.6%) in the spontaneous labor group, respectively (peripartum major bleeding: OR 4.90, 95% CI 0.44–54.79, *p* = 0.21; peripartum minor bleeding: OR 4.21, 95% CI 1.57–11.31, *p* = 0.002). Among patients who did not receive LMWH during pregnancy, four (6.2%) had peripartum major bleeding and seven (0.8%) had peripartum minor bleeding in the induction of labor group, compared to one (0.6%) and nine (5.3%) in the spontaneous labor group, respectively (*p* = 0.02 peripartum major bleeding, *p* = 0.15 peripartum minor bleeding). There were no postpartum VTE events or maternal mortalities reported. There were six neonatal deaths, one (0.7%) among a woman who received an induction of labor and five (1.4%) with spontaneous labor (Table 3). Of the six neonatal deaths, four were delivered preterm (36 weeks, 34 weeks, 28 weeks and 24 weeks), four were delivered by emergency CD, and all were SGA (1 < 3rd percentile; 3 < 5th percentile; 2 < 10th percentile). Women who delivered these infants had a previous history of pre-eclampsia in four of the cases, and confirmed pre-eclampsia during the current AFFIRM pregnancy in two of the six cases.

## Discussion

There was no increased risk of CD among women with past placenta-mediated pregnancy complication who underwent induction of labor, compared to spontaneous labor. Our data adds to a reassuring and growing body of literature that suggests there is no increased CD risk based on these indications for induction [12–16]. While our results are not generalizable to a low-risk obstetrical population, they support the results of the randomized ARRIVE trial that showed a decreased risk of CD among low-risk nulliparous women randomized to induction of labor (18.6% versus 22.2%, RR 0.84, 95% CI 0.76–0.93) [10], with a difference in CD rates that persisted across the pre-specified subgroups of age, ethnicity, body mass index and modified Bishop score.

An important subgroup where induction of labor had a lower risk of CD in our study was women who were randomized to receive LMWH during their pregnancies. These results suggest that women receiving LMWH during pregnancy may benefit from induction of labor to reduce the risk of CD. The strength of this subgroup analysis is that LMWH use was randomized. It is possible that the group of women who received LMWH may have had closer monitoring during pregnancy compared to women without LMWH, which could have resulted in improved delivery outcomes. Details such as the timing of LMWH use during pregnancy and epidural anesthesia rates by group are unknown. LMWH during pregnancy was used in the context of a clinical trial to prevent recurrent placenta-mediated pregnancy complications, so LMWH use may have been stopped days to weeks prior to labor and delivery according to the individual trial protocol. There was no difference in gestational age of delivery between the LMWH group and the no LMWH group.

There was an increased risk of peripartum major and minor bleeding after induction of labor, compared to spontaneous labor. In the absence of an increased risk of CD, this data is hypothesis-generating and requires confirmatory studies. Previous studies report only the postpartum bleeding risk, which is based on variable study definitions that often do not capture immediate peripartum bleeding. Meta-analyses evaluating elective induction of labor versus expectant management have reported no increase in postpartum hemorrhage [3], or insufficient evidence of postpartum hemorrhage based on a limited number of studies [2]. Additionally, there was no increased rate of postpartum hemorrhage in a randomized trial evaluating induction of labor among women with pre-eclampsia [12]. One major strength of our study was that bleeding was an outcome of interest in all included clinical trials, and individual patient data was recoded based on a common definition of peripartum major bleeding, peripartum minor bleeding, and postpartum (> 24 h) bleeding. However, how the actual blood loss was estimated likely varied across sites. Unfortunately, we do not have data on the type of induction methods used, reason for induction, or the rates of operative vaginal delivery between the two groups, which could theoretically affect the bleeding risk.

There are several limitations to our study. We evaluated labor and delivery outcomes among randomized trials that evaluated the use of LMWH, so the groups of women who underwent induction of labor and spontaneous labor were not randomized and so selection bias could have been present. Because our study was non-randomized, there are differences in baseline characteristics between groups such as mean gestational age, and certain patient characteristics may have influenced the decision for induction of labor (Table 1). Based on our available data we compared induction of labor to spontaneous labor instead of expectant management, whereas expectant management is actually the real decision that clinicians face. We excluded women who underwent an unplanned or emergency CD without a trial of labor; however, if we had included these deliveries (that did not undergo labor induction) then it is possible that our results would have further favored the induction of labor group.

While there are differences in the proportion of induction of labor versus spontaneous labor in patient subgroups (Table 1), we cannot draw any conclusions because the individual trials had different inclusion criteria and may have had regional differences in their induction of labor practices (Table 2). We excluded all pregnancy losses, but acknowledge that an early pregnancy loss and a late fetal loss are managed differently, with the latter including possible induction of labor. Our study did not collect important information that would give us a complete picture of CD risk, such as previous CD rates, reason for induction of labor, reason for failed induction, previous induction of labor rates or cervical status based on the Bishop score. Additionally, important outcomes that were missing in our study included operative vaginal delivery, maternal infections, wound complications, and neonatal outcomes such as APGAR scores, meconium-stained amniotic fluid, meconium aspiration syndrome, and admission to a neonatal intensive care. We also had small subgroups of patients, so further studies are still needed to evaluate specific subgroups such as patients with pre-eclampsia.

## Conclusion

In summary, we provide additional evidence that there is no increased risk of CD after induction of labor among a high-risk population of patients with previous placenta-mediated pregnancy complications. Our results suggest that women who receive prophylactic LMWH during pregnancy might benefit from an induction of labor. Further research is still needed to confirm the risks of induction of labor among higher-risk patients with placenta-mediated pregnancy complications.

## Data Availability

It is not possible to obtain the anonymized data. The datasets generated and analysed are not publicly available.

## Abbreviations

BMI: Body mass index
CD: Cesarean delivery
CTPA: Computed tomography pulmonary angiography
DVT: Deep vein thrombosis
LMWH: Low-molecular-weight heparin
PE: Pulmonary embolism
SD: Standard deviation
SGA: Small for gestational age
VTE: Venous thromboembolism
